# *In situ* repair of bone and cartilage defects using 3D scanning and 3D printing

**DOI:** 10.1038/s41598-017-10060-3

**Published:** 2017-08-25

**Authors:** Lan Li, Fei Yu, Jianping Shi, Sheng Shen, Huajian Teng, Jiquan Yang, Xingsong Wang, Qing Jiang

**Affiliations:** 1 School of Mechanical Engineering, Southeast University, Nanjing City, Jiangsu Sheng China; 2 Department of Sports Medicine and Adult Reconstructive Surgery, Drum Tower Hospital affiliated to Medical School of Nanjing University, Jiangsu Sheng, China; 3 Drum Tower of Clinical Medicine, Nanjing Medical University, Jiangsu Sheng, China; 4 Laboratory for Bone and Joint Diseases, Model Animal Research Center, Nanjing University, Jiangsu Sheng, China; 5 School of Electrical and Automation Engineering, Nanjing Normal University, Jiangsu Sheng, China

## Abstract

Three-dimensional (3D) printing is a rapidly emerging technology that promises to transform tissue engineering into a commercially successful biomedical industry. However, the use of robotic bioprinters alone is not sufficient for disease treatment. This study aimed to report the combined application of 3D scanning and 3D printing for treating bone and cartilage defects. Three different kinds of defect models were created to mimic three orthopedic diseases: large segmental defects of long bones, free-form fracture of femoral condyle, and International Cartilage Repair Society grade IV chondral lesion. Feasibility of *in situ* 3D bioprinting for these diseases was explored. The 3D digital models of samples with defects and corresponding healthy parts were obtained using high-resolution 3D scanning. The Boolean operation was used to achieve the shape of the defects, and then the target geometries were imported in a 3D bioprinter. Two kinds of photopolymerized hydrogels were synthesized as bioinks. Finally, the defects of bone and cartilage were restored perfectly *in situ* using 3D bioprinting. The results of this study suggested that 3D scanning and 3D bioprinting could provide another strategy for tissue engineering and regenerative medicine.

## Introduction

Three-dimensional (3D) printing, known as additive manufacturing, has led to a grand revolution in medicine and life sciences. The placement of cells, biomaterials, and biomolecules is extremely precise in spatially predefined locations through computer-aided design (CAD) and computer-aided manufacturing (CAM)^1^. It might be the therapeutic prospect of some diseases and transform the concept of traditional tissue engineering and regenerative medicine. The advances in the field of tissue engineering today allow the fabrication of more and more complex 3D constructs, containing multiple cell types, extracellular matrices, and bioactive stimuli^2^. Recent studies have reported the progress in constructing tissues and organs, including heart valve, cartilage, bone myocardial tissue, trachea, and blood vessels^3–7^. Researchers firmly believe that 3D printing has the ability to overcome some of the engineering challenges encountered in the field of regenerative medicine and tissue engineering.

The current strategy of scaffold-associated therapy is to culture cells in a scaffold to create a mature tissue *in vitro* prior to implantation^8^. Different additive manufacturing techniques, such as inkjet printing, bioextrusion, laser-based printing, and photopolymerization, have been developed to fabricate scaffolds for tissue engineering applications^1, 9^. The ideal scaffolds or substitution for tissue engineering must meet several requirements, including biocompatibility, biodegradability, and porosity, but the most important thing is the exact shape to fit the damaged area. A series of advancements has been made, such as vascularized cell-laden constructs^10^, cartilaginous tissues containing poly(ethylene glycol)/hyaluronic acid and chondrocytes^11^, and bone regeneration scaffolds made up of calcium phosphate and coated with collagen^12^. The research emphases are on the structure and forming process of scaffolds and the synthesis and modification of biomaterials. Undoubtedly speaking, 3D bioprinting strategies have demonstrated their ability in fabricating scaffolds with multiple biomaterials and cells. The scaffolds used for regenerative therapies are expected to integrate with existing native tissue and repair lesions of different sizes and thicknesses. The 3D printing, especially 3D bioprinting, has been regarded as a potential and powerful tool to reconstruct tissue and organ structures after the injuries were included.

However, few studies have focused on the role of 3D printing in morphological repair *in situ*. In effect, fabricating customizable implants *in situ* by 3D bioprinting is difficult and challenging. Obtaining the shape of defects, synthesizing appropriate biomaterials, printing customized-form hydrogel in a short time, and using a specific 3D printer to realize this process are necessary conditions to achieve this goal. The aforementioned status may be the reason why 3D bioprinting is not yet widely used in this field.

This study demonstrated the *in situ* repair of a cuboid-shaped bone defect, a random-shaped cartilage defect, and a cylindrical-shaped osteochondral defect. Alginate hydrogel was used as a bioink for the repair of bone and osteochondral defect because it has been thoroughly investigated to produce biocompatible, osteoconductive scaffolds suitable for bone repair. A modified sodium hyaluronic acid (HA) based hydrogel was used to repair the cartilage defect. HA has many unique properties, including its biocompatibility, viscoelasticity, and lack of immunogenicity. Furthermore, HA is one of the major ingredients of cartilage extracellular matrix (ECM) and is involved in cell proliferation, morphogenesis, inflammation, and wound repair. This kind of modified photopolymerizable HA hydrogel showed degradation properties and chondrogenic ability in a previous study, which strongly supported its suitability in cartilage tissue engineering^13^. A combination of modified HA hydrogel and a kind of small molecular compound demonstrated the favorable capacity of cell homing and cartilage repairing.

**Figure 1:**
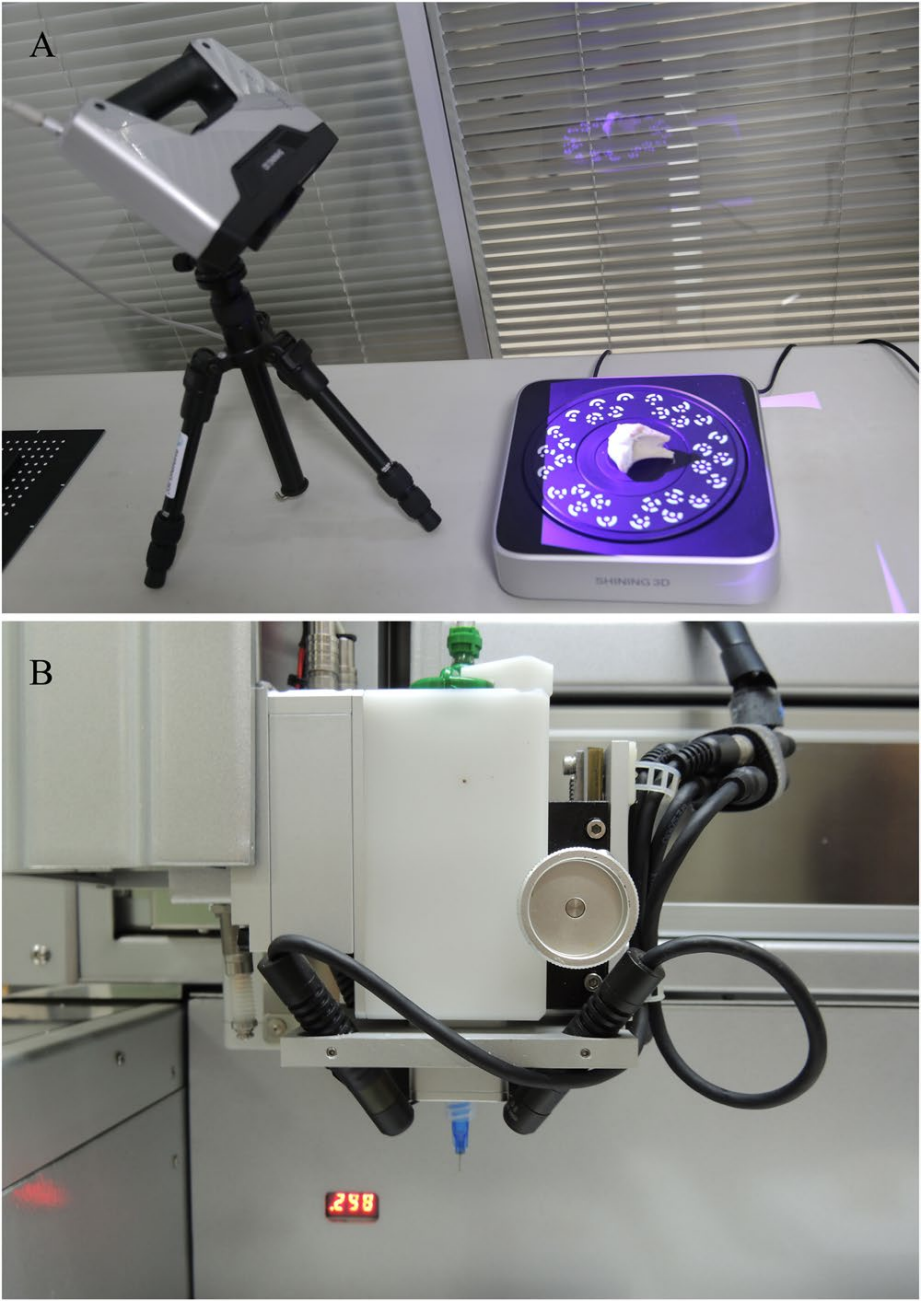
Process of 3D scanning and nozzle of the 3D bioprinter. (**A**) 3D scanning process of tibial plateau. We have been authorized by the owner to use the logo in this figure. (**B**) Nozzle of the 3D bioprinter was modified with four long-wave UV lights.

The objective of this study was to test the feasibility of 3D scanning and 3D bioprinting to precisely restore the lesion shape (Fig. 1A,B). The criteria for success were to make suited CAD models, achieve control placement, and form a hydrogel. The fundamental instrument, modeling method, and fabrication parameters provided in this study might assist the adoption of 3D bioprinting technologies for the development of individual and precision medical model in the future.

## Results

### 3D scanning

**Figure 2:**
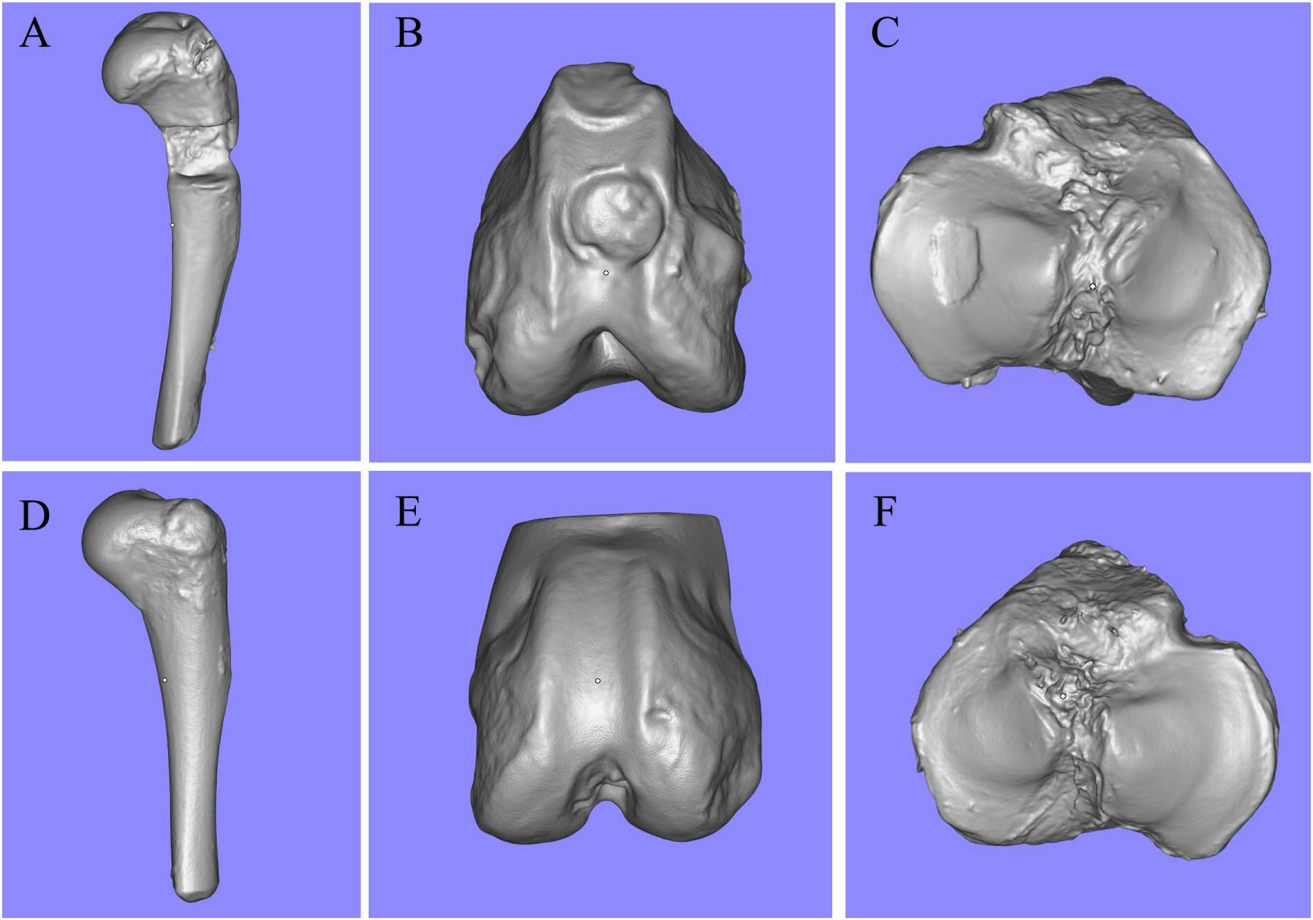
Digital models of six samples obtained by 3D handheld scanner. (**A**,**D**) Bone defect model and a healthy sample of the contralateral side. (**B**,**E**) Osteochondral defect model and a healthy sample of the contralateral side. (**C**,**F**) Chondral defect model and a healthy sample of the contralateral side.

**Figure 3:**
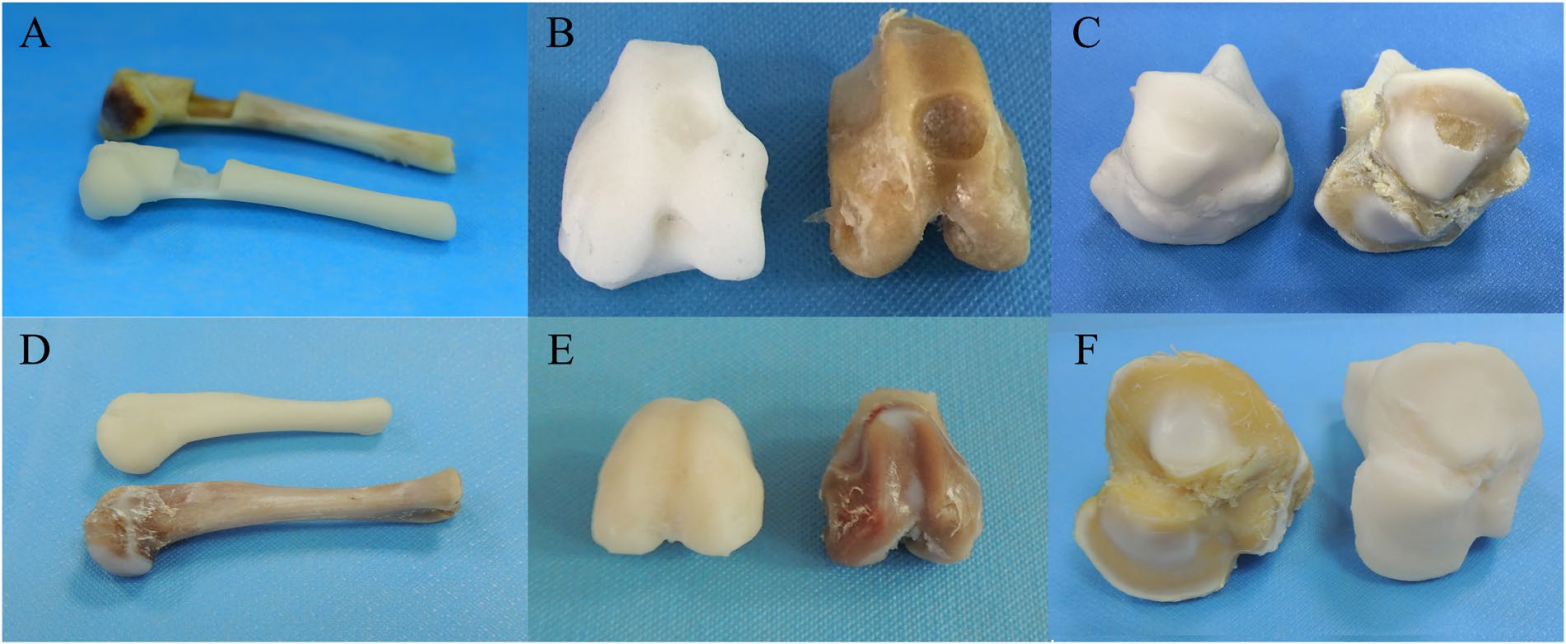
Photosensitive resin models of scanning data. (**A**,**D**) Comparison of original humeral bones and resin models. (**B**,**E**) Comparison of femoral condyles and resin models. (**C**,**F**) Comparison of tibial plateau and resin models.

Each sample was 3D scanned from three different angles to ensure that all surfaces of the samples were captured by the camera. It took about 5 minutes to complete the scanning process. Scanning data were exported to STereo Lithography (STL) format and restored using the Magics software (Fig. 2). A comparison and contrast between the origin samples and resin models demonstrated that the morphological specificities of the surface of injury or health were recreated accurately (Fig. 3).

### Bone defect

**Figure 4:**
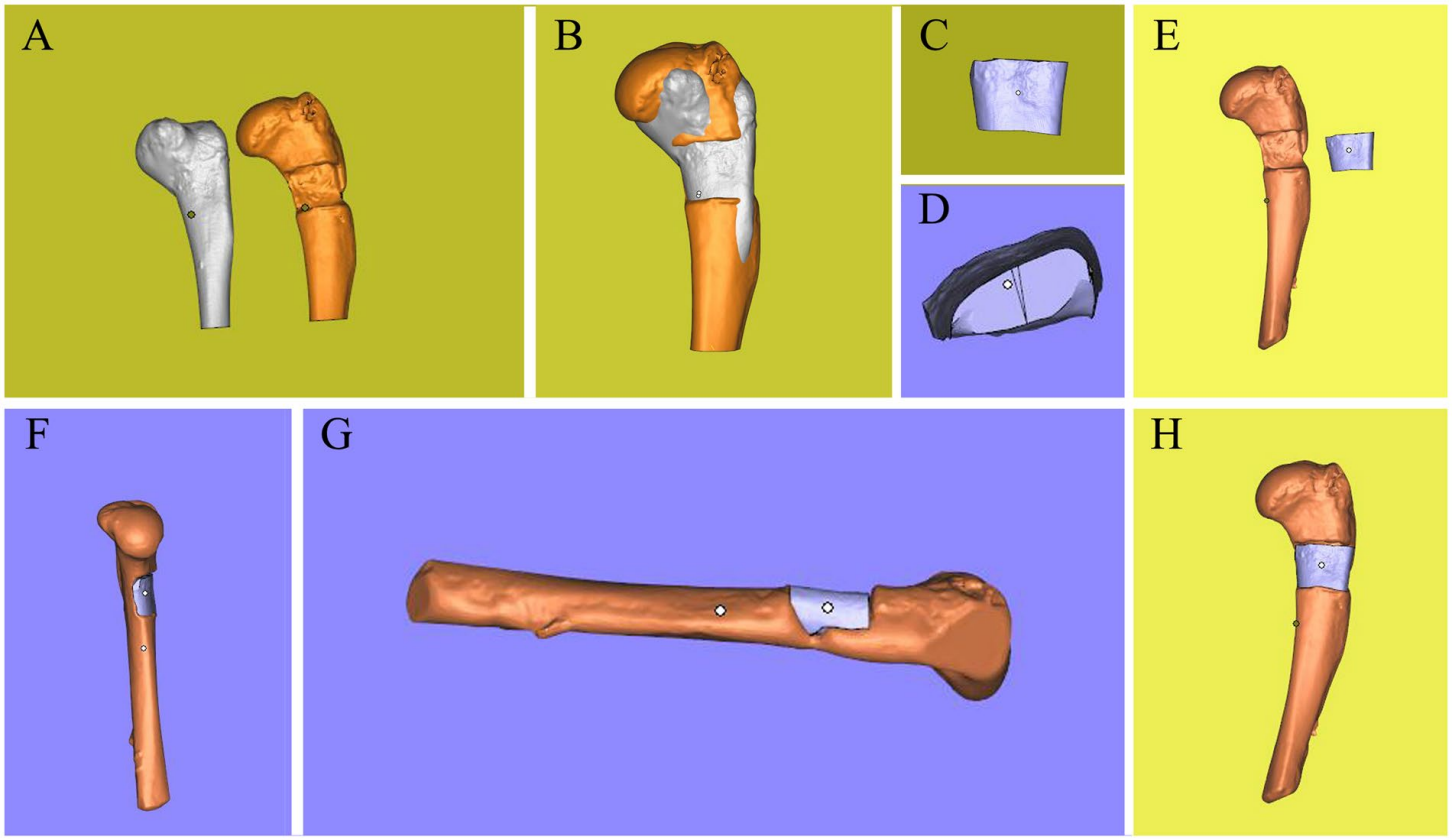
Three-dimensional digital models of humeral bones. (**A**) Humeral bone digital models with and without defect. (**B**) Matching of two digital models and using Boolean operation. (**C**) Frontal view of printing geometry. (**D**) Lateral view of printing geometry. (**E**) Frontal view of bone defect model and printing geometry. (**F**) Left side view of assembly drawing. (**G**) Right side view of assembly drawing. (**H**) Frontal view of assembly drawing.

The 3D scans from damaged humeral bone and healthy corresponding part were processed and subtracted using the Magics software and Boolean operation to build a model of the defect (i.e. the target printing geometry) (Fig. 4A,B). The lateral view of geometry was a trapezoid, and the anterior view was a rectangle (Fig. 4C,D). The volume of defect was 242.02 mm^3^. The 3D geometry model and bone defect model were assembled in the Magics software to test the geometric fidelity (Fig. 4E–H).

**Figure 5:**
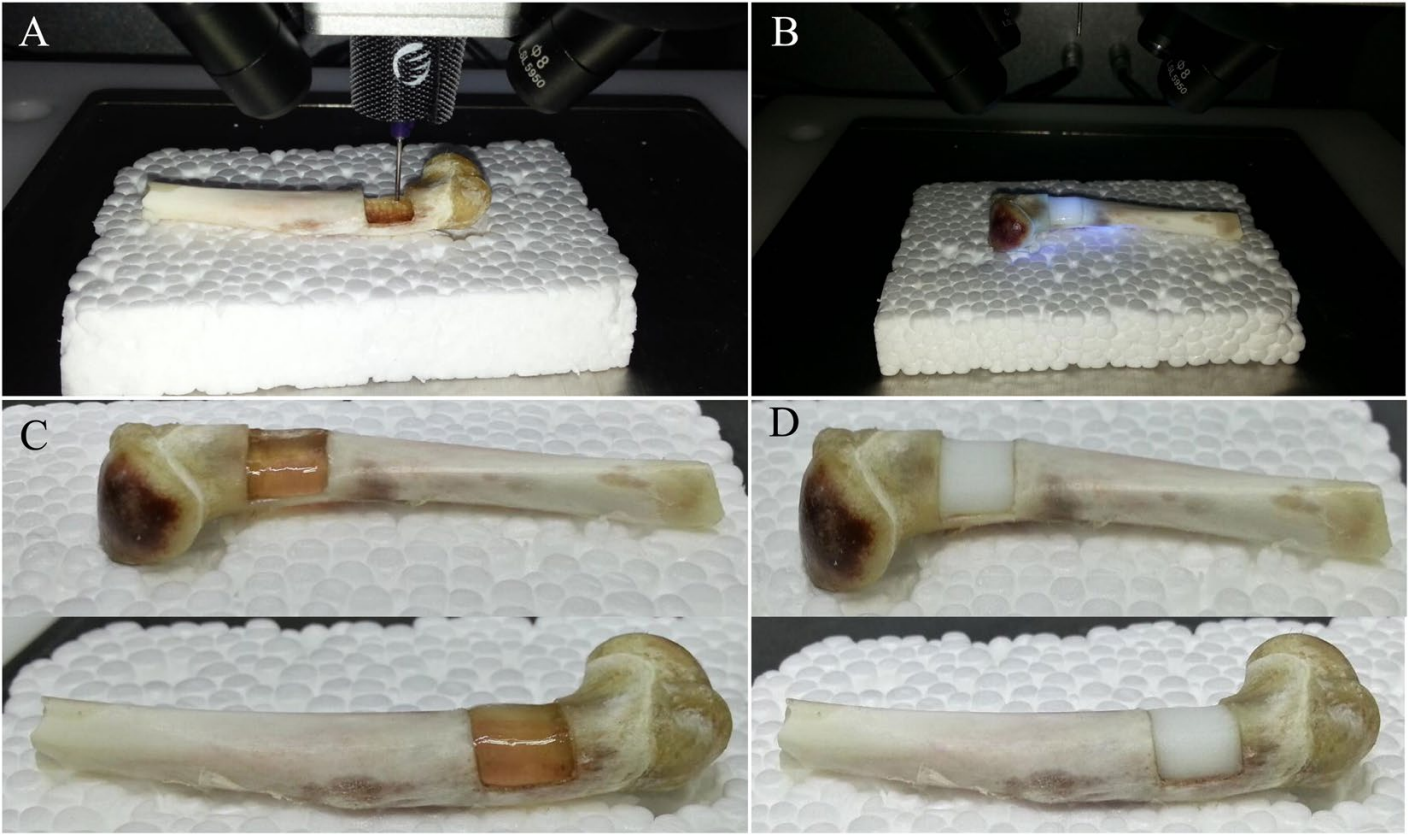
Process of 3D bioprinting and photopolymerization on bone defect. Due to the influence of UV light of camera, the light was turned off when shooting video and photographs in one of the three printing process. Photopolymerization was taken at the end of printing. (**A**) Repair of bone defect through *in situ* 3D bioprinting with alginate hydrogel. (**B**) Exposure to UV light. (**C**) Alginate hydrogel that was printed to repair the bone defect was transparent before photopolymerization. (**D**) The color of alginate hydrogel turned milky white after being exposing to UV light in few seconds. The bone defect was restored perfectly.

The printing parameters for alginate were determined through its viscosity and photo-crosslinking time. The entire printing process was exposed to UV light for photopolymerization (Fig. 5A,B). In this way, hydrogel could maintain a stable structure to match the void present in the humeral bone (Fig. 5C,D). The alginate hydrogel construct was printed directly into the defect for three times to verify the repeatability of this operation. The mean time of the total printing process was 120.91 s. The surface and sides of the printed structure were closely matched defect according the three prints.

### Osteochondral defect

**Figure 6:**
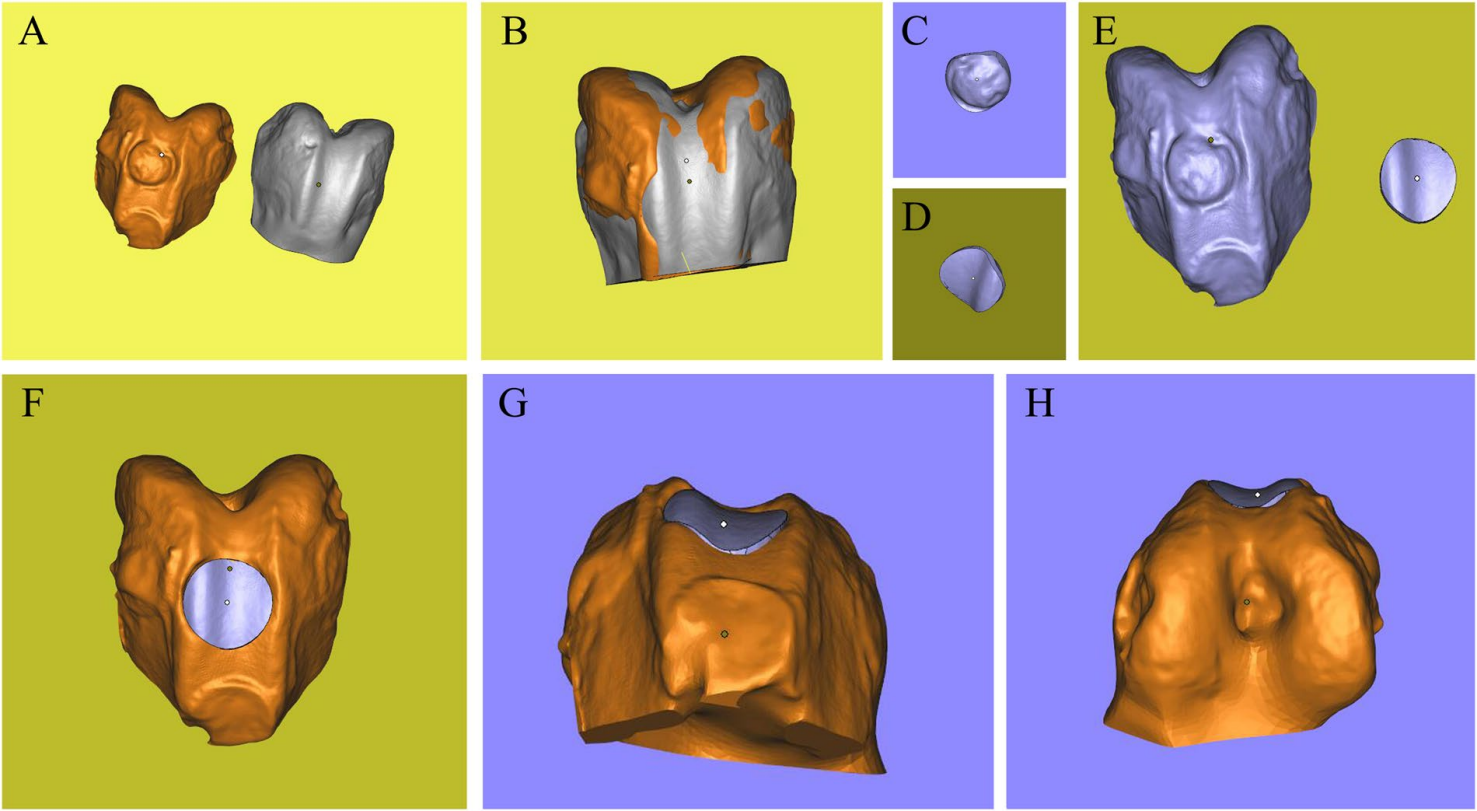
Three-dimensional digital models of femoral condyle. (**A**) Femoral condyle digital models with and without defect. (**B**) Matching of two digital models and using Boolean operation. (**C**) Posterior view of printing geometry. (**D**) Frontal view of printing geometry. (**E**) Frontal view of osteochondral defect model and printing geometry. (**F**) Frontal view of assembly drawing. (**G**) Posterior view of assem bly drawing. (**H**) Anterior view of assembly drawing.

As in bone defect, the 3D scaning from damaged femoral condyle and the corresponding healthy part were processed to build the target printing geometry (Fig. 6A,B). This geometry comprised two structures: a dome-shaped bone covered by a cylindrical cartilage cap (Fig. 6C,D). The volume of the defect was 45.34 mm^3^. Alginate hydrogel was again used for repairing this defect, and the same printer parameters as in the bone defect case were used. Printing operation was repeated for three times, and the mean time of whole printing process was 24.73 s.

**Figure 7:**
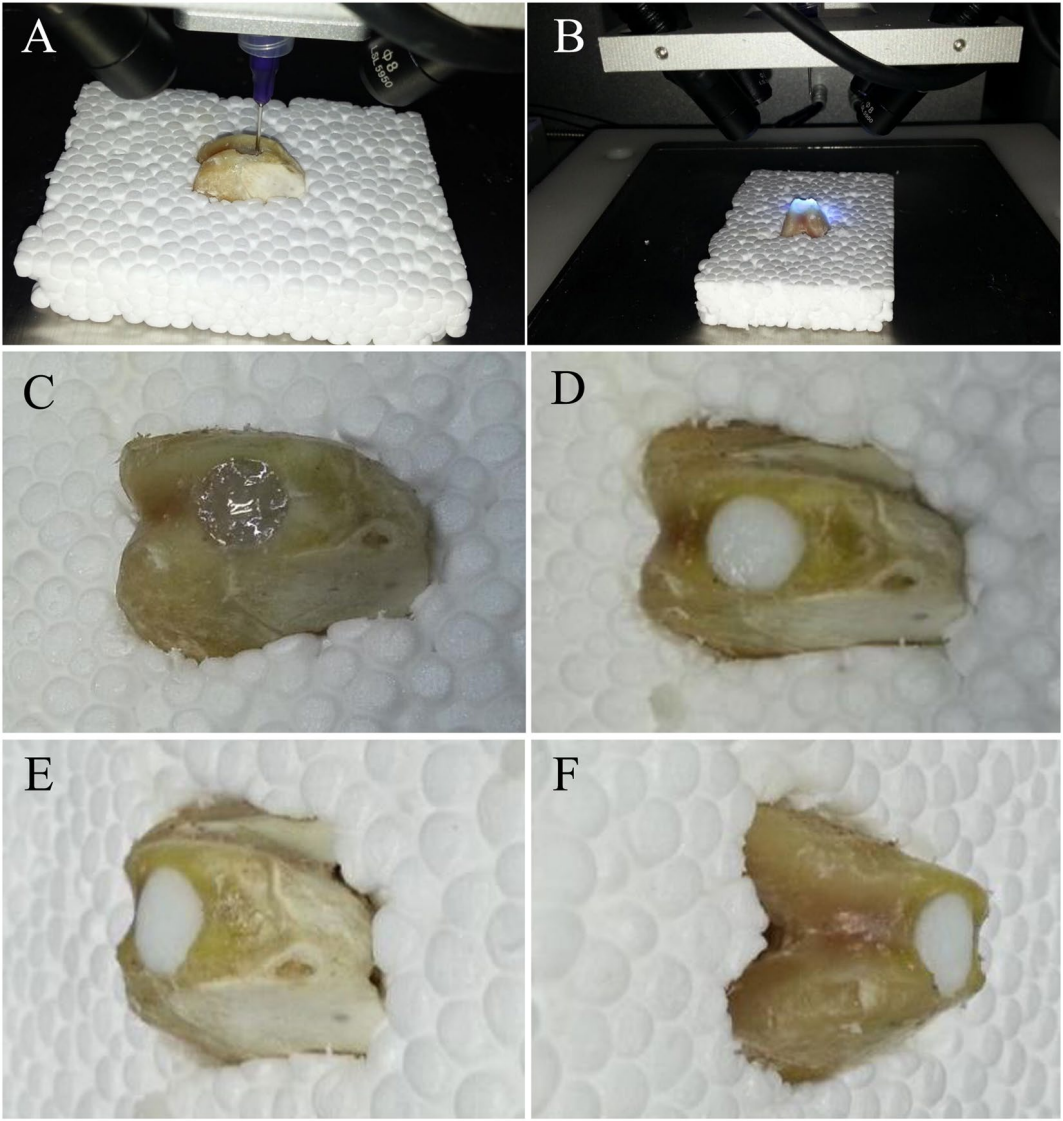
Process of 3D bioprinting and photopolymerization on osteochondral defect. Due to the influence of UV light of camera, the light was turned off when shooting video and photographs in one of the three printing process. Photopolymerization was taken at the end of printing. (**A**) Repair of osteochondral defect through *in situ* 3D bioprinting with alginate hydrogel. (**B**) Exposure to UV light. (**C**) Alginate hydrogel that was printed to repair the osteochondral defect was transparent before photopolymerization. (**D**–**F**) The color of alginate hydrogel turned milky white after being exposing to UV light in few seconds. The cartilage cap was slightly higher than the contour of the condyle according to the three views of samples.

According to the assembling images (Fig. 6E–H) and the effect of restoration (Fig. 7), the cartilage cap was slightly higher than the contour of the condyle. This was caused by the fact that the geometric fidelity of damaged femoral condyle and corresponding healthy femoral condyle could not match perfectly.

### Chondral defect

**Figure 8:**
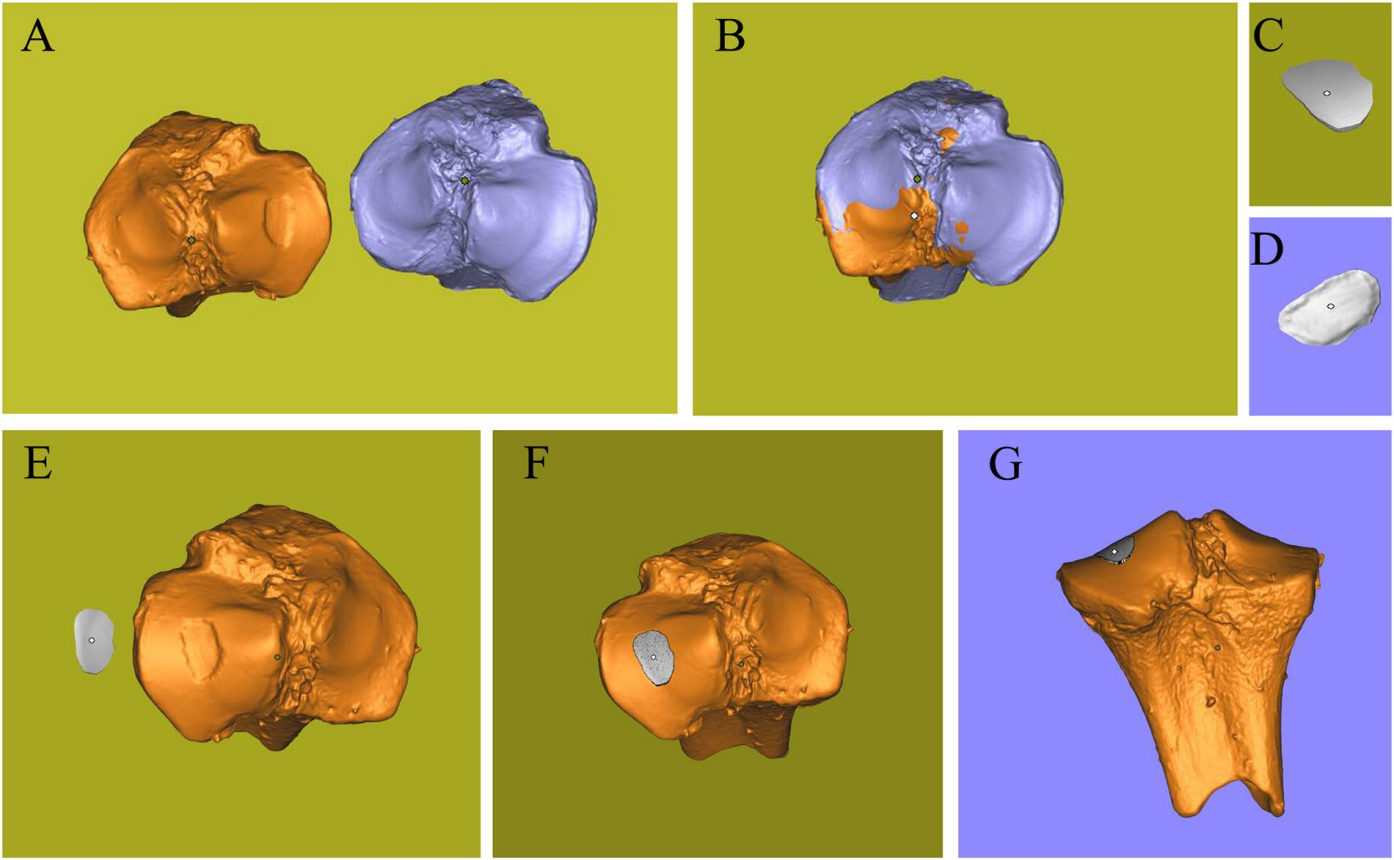
Three-dimensional digital models of tibial plateau. (**A**) Tibial plateau models with and without defect. (**B**) Matching of two digital models and using Boolean operation. (**C**) Frontal view of printing geometry. (**D**) Posterior view of printing geometry. (**E**) Frontal view of bone defect model and printing geometry. (**F**) Frontal view of assembly drawing. (**G**) Anterior view of assembly drawing.

**Figure 9:**
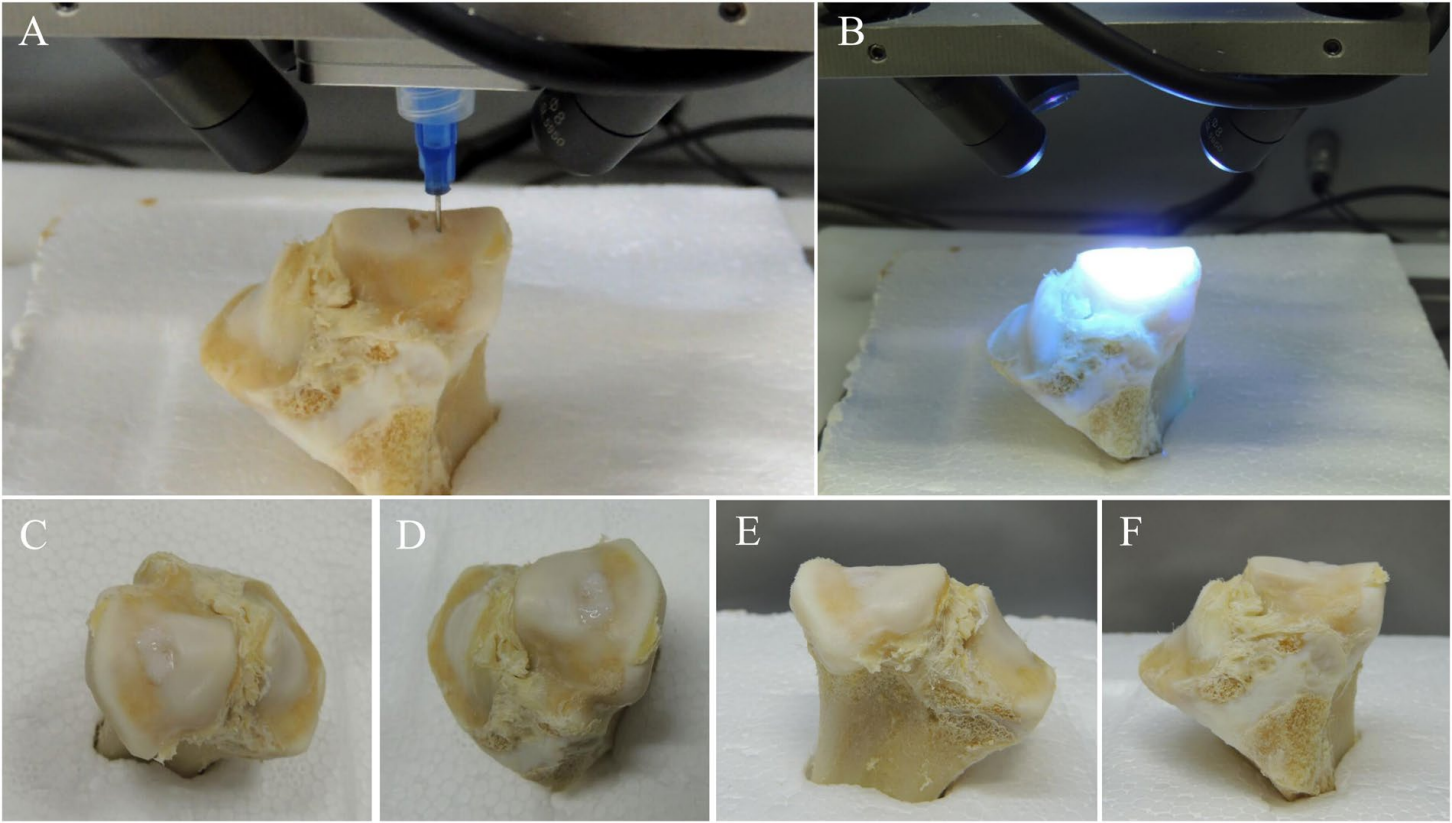
Process of 3D bioprinting and photopolymerization on chondral defect. Due to the influence of UV light of camera, the light was turned off when shooting video and photographs in one of the three printing process. Photopolymerization was taken at the end of printing. (**A**) Repair of chondral defect through *in situ* 3D bioprinting with m-HA hydrogel. (**B**) Exposure to UV light. (**C**–**F**) The color of m-HA hydrogel that was printed to repair the chondral defect was milky white before and after photopolymerization. The chondral defect was restored perfectly.

Digital models were processed as described previously (Fig. 8A,B). The geometry was an ellipse with 9 mm length and 6 mm width (Fig. 8C,D). The volume of defect was 39.29 mm^3^. The printing parameters were modified to adapt to the viscosity of HA. The air pressure was enlarged, and nozzle speed was slowed down to maintain an even diameter of the hydrogel filament. The printed geometry closely matched the intended surface contour of the substrate’s cartilage tissue (Fig. 9), and the mean time of the whole printing process completed was 36.61 s.

### Accuracy measurement

**Figure 10:**
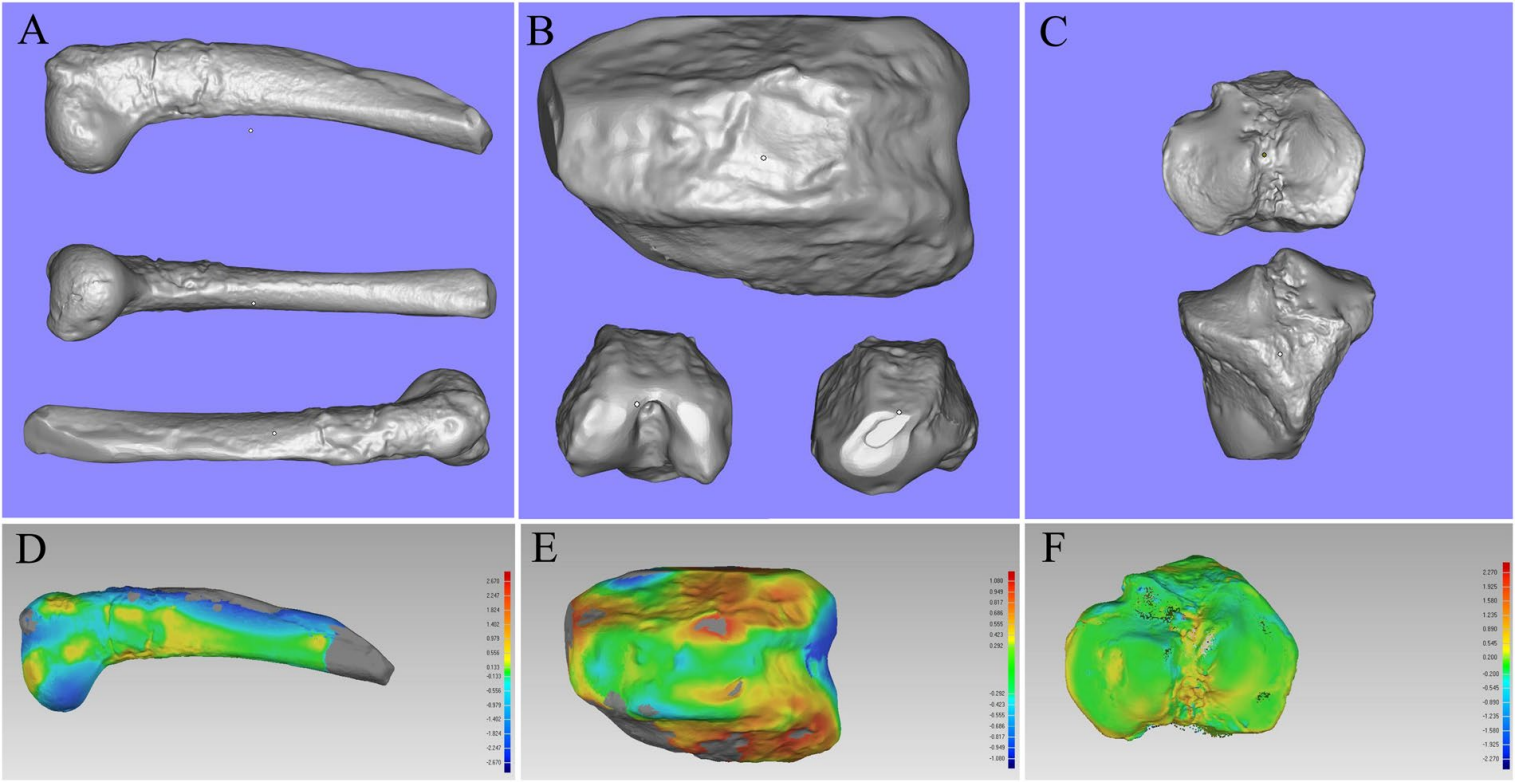
Measurement of *in situ* 3D bioprinting. (**A**) 3D model of the repaired bone defect. (**B**) 3D model of the repaired osteochondral defect. (**C**) 3D model of the repaired chondral defect. (**D**) Result of 3D Samples Comparison on bone defect. The major color on the surface of were green and yellow, and the partial boundary was blue. (**E**) Result of 3D Samples Comparison on osteochondral defect. The major color on the surface of was green, and the bilateral boundary were yellow and light red. (**F**) Result of 3D Samples Comparison on chondral defect. The major color on the surface of were green and yellow. The boundary was green and the central region was yellow.

The accuracy was measured using two kinds of professional software: Geomagic Qualify and 3-matic STL. The results of 3D Samples Comparison are shown in Fig. 10. The 3D error between the surface of printed samples and corresponding healthy parts was shown in Table 1 as mean ± standard deviation. Different colors represent the tolerance and fidelity of printed surface. On the printed surfaces of the three samples of both, the major colors were yellow and green. It meant that the geometric morphology of printed surfaces was fairly accurate to healthy parts. In the model of bone and cartilage defect, the height of hydrogel implant was the same as the rest part. For the osteochondral defect, the edge of implant was about 0.2 mm higher than the surrounding area.

**Table 1:** Printing fidelity.

	Bone Defect	Osteochondral Defect	Chondral Defect
Mean Error (mm)	0.322 ± 0.696	0.458 ± 0.322	0.303 ± 0.223

## Discussion

Three models were created in this study to mimic three orthopedic diseases: large segmental defects of long bones, freeform fracture of femoral condyle and ICRS grade IV chondral lesion. The feasibility of *in situ* 3D bioprinting for these diseases was explored.

According to the 3D samples comparison, the three defects could be repaired satisfactorily using 3D bioprinting. Two different hydrogels were chosen to repair bone and cartilage, respectively. Alginate hydrogels were probably the most widely used biomaterials in 3D bioprinting because of its biocompatibility, reversible control over stiffness, and capability to form highly porous structures for cell regeneration^14, 15^. The biocompatible materials sodium alginate and PEGDA were chosen in this study to constitute an interpenetrating network. Alginate and poly(ethylene glycol) (PEG) polymers are ionically and covalently cross-linked through Ca^2+^ and UV exposure, respectively. The alginate chains are detached from the reversible ionic crosslinks when the hydrogel is deformed. Once the hydrogel is relaxed from deformation, it reverts to its original configuration as the covalently cross-linked PEG network maintains the elasticity of the hydrogel^16–18^. Due to these characteristics, the alginate-PEGDA hydrogel is highly stretchable and tough to provide sufficient strength and stiffness to function for a period until bone and cartilage remodeling.

In addition to sodium alginate, HA-based materials are emerging as a cell scaffold platform for tissue engineering applications, including the creation of tissue-engineered bone of cartilage. They can stimulate chondrocyte metabolism, synthesize cartilage matrix components, and inhibit chondrodegenerative enzymes as well as inflammatory process^19, 20^. As intra-articular-injected material, they also possess a high swelling ratio needed to cover the articular surface^19^. HA was modified to form photopolymerizable hydrogels with MA in this study. Photopolymerization occurred under relatively mild conditions and allowed the facile fabrication of complex shapes. The physical properties of m-HA hydrogels were easily altered by changing either the degree of HA modification or through copolymerization with photopolymerizable macromolecules^21, 22^. This ability to tailor the properties of m-HA hydrogel allowed great flexibility to meet those needed for cartilage repair.

Suitable biomaterials and stimulating factors, which could recruit stem cells from bone marrow and synovialis^13, 23^, are important component of bioink for 3D bioprinting. Several studies confirmed that alginate combined with bone morphogenetic protein 2 (BMP-2), collagen, and oligosaccharides plays vital roles in bone cell proliferation, migration, and differentiation^24–28^. HA hydrogel with different molecular weight could facilitate cell migration and modulate cell proliferation^29–31^. These studies provided strong evidences for the migration of cells in alginate and HA hydrogel. For cell *in vivo* migration, dense connective tissue, loose connective tissue, and tightly packed basement membrane were the three major types of microenvironment^32^. Suitable biomaterials could mimic these environments and affect the ability of cell migration. Hydrogels were pliable enough for cells, which have the ability to sense areas of increased stiffness and move preferentially towards them^33^. It was entirely possible for cell migration in biomaterials *in vivo*. Cross-linking of hydrogels can be catalyzed by varying temperature and pH, as well as exposure to UV light during the printing. In a previous study, kartogenin (KGN), a small-molecular-weight compound, was encapsulated in the poly(lactic-co-glycolic acid) (PLGA) nanoparticles to make a drug-delivery system. The PLGA nanoparticles were mixed with m-HA to form an injectable hydrogel. The regenerated cartilage induced by this hydrogel was close to natural hyaline cartilage. The proportion ratio of m-HA was changed in this experiment to verify its potential of 3D bioprinting. It laid the foundation for future experiments on animals. Some other kinds of nanoparticles wrapped with BMP-2, isopsoralen, and glucosamine sulfate for bone and cartilage repair were tested in other studies. A combination of drug and/or growth factors with bio-ink for *in situ* 3D printing might be an excellent alternative for injury treatment.

Each step of the experimental process was critical. The samples were put on a platform that was capable of automatically rotating from 0° to 360° to acquire high-resolution digital models. The scanner remained immobile as the samples were revolving during the scanning process. This way, every detail could be acquired by the 3D scanner. For the purpose of completing the process quickly and precisely, the operator needed to practice repeatedly, especially in handheld mode, which provided a more convenient scanning method to reduce the scanning time without losing accuracy.

The extrusion-based 3D bioprinting/photopolymerization method provided a preliminary attempt of computer-controlled layer-by-layer construction of different hydrogel for bone and cartilage repair *in situ*. The difficulties to print *in situ* were recognition of print plane and placement degree of digital models. Different from printing on the base, the 3D printer could not determine the print plane by identifying three points on it. The print plane in this study was determined by recognizing the height between the nozzle and the lowest point of defect. However, the placement degree of digital models determined the starting point and printing path in this system. Some preliminary experiments were taken to confirm the correlation between placement degree in computer and printing objectives on base. Finally, the customized hydrogel was successfully printed in the defect.

The printing resolution in this study (layer thickness, 180 μm) was higher than that of the previously reported method of *in situ* printing of chondral defect using syringe-extruded alginate hydrogel (layer thickness, 800 μm)^34^ but less than that of the method of inkjet-based *in situ* printing using poly(ethylene glycol) hydrogel (layer thickness, 85 μm)^35^. However, resolution in the present study still had room for improvement, composition proportion of alginate hydrogel and m-HA hydrogel could be adjusted to achieve more appropriate viscosity and photo-corsslinking time. The proportion of alginate and m-HA were not specific in this study. For the sake of ensuring success rate and repeatability, the most commonly used proportion ratio was chosen in this study as the candidate. Photo-polymerization was chosen as the shaping method for the same reason. It was fast and convenient, and no post-processing. The effects on resolution can be developed by differing the proportion ratio of hydrogel in the future studies. The viscosity of bio-ink was related to the proportion of hydrogel, including polymer solvent miscibility, polymer molecular weight, and polymer concentration, which determined the printing parameters and cell behavior^36, 37^. In addition, proportion of hydrogel also determined the material flow rate, printed line height, and linear write speed. Almost all of the printing parameters have to be adjusted with the change of hydrogel proportion. As the increase of viscosity, more air pressure was needed to squeeze out the bio-ink. When the viscosity was high and the printing speed was slow, the shape of printed objects could be better maintained. However, the cell viability would decrease significantly under the high viscosity microenvironment^38^. Searching a balance hydrogel proportion between printing and maintaining cell viability was an indispensable part of 3D bioprinting.

3D scanning and 3D bioprinting are promising methods for treating open injuries in the skeletal system. The resolution of 3D scan can be up to 5 μm, which is particularly attractive for reestablishing the articular cartilage superficial zone (only about 200 μm thick and almost impossible to repair manually)^39^. The instrument itself is portable and convenient to operate; operators can obtain precise 3D digital model in few minutes in emergency room or operating room. The total process, including 3D scanning and 3D bioprinting, can be completed in 10–15 min in the present study. In some cases, this procedure is cheap, radiationless, and effective compared with computed tomography (CT) and magnetic resonance imaging (MRI). Formation of entities can be achieved through 3D printing. Thus, an ideal biomaterial structure is fabricated *in situ* to provide mechanical support locally and deliver growth factors to support tissue growth.

Bone healing and reconstruction after a variety of etiologies are processes involving a series of cellular and mechanical events culminating in the reestablishment of bone integrity^40^. Vascularized bone graft has emerged as gold standards in the last 40 years. Various donor sites, such as iliac crest, ribs, and fibula with accompanying skin paddles or muscle components, can be used for vascularized bone graft^41^. For the reconstruction of massive bony defects, patients undergo several operations, and the postoperative complications are usually beset with a range of consequences for patients^42^. Two novel techniques have emerged recently: the Masquelet technique and the Cylindrical Titanium Mesh Cage (CTMC) with polylactide membranes technique^43–45^. Osteogenic proteins are usually combined with different carrier materials for clinical use^46–48^. Recently, osteogenic cells, growth factors, and biomaterial scaffolds form the foundation of numerous bone tissue engineering strategies^40^.

Chondral and osteochondral lesion, resulting from osteoarthritis, aging, and joint injury, are a major cause of joint pain and chronic disability^35^. Mature cartilage cannot heal spontaneously without blood vessels, nerves and lymphatics^11^. Arthroscopic microfracture, chondrocyte transplantation, autologous osteochondral transplantation and mosaicplasty are common therapeutic methods for ICRS grade III-IV chondral defects^49^. For advanced cartilage degeneration, joint replacement surgery is the most common treatment^50–52^. It is also highly invasive, complicated and expensive. Although cell transplantation-based tissue engineering treatment for human cartilage repair was introduced almost two decades ago, current cartilage tissue engineering strategies cannot fabricate new tissue that is indistinguishable from native cartilage with respect to zonal organization, extracellular matrix composition and mechanical properties^23, 53, 54^.

## Conclusions

The bridging of a large segmental bone defect and osteocondroal defect requires a plan that takes into account the affected part, the etiologies and its specific requirements, the patient’s physiological/psychological state and expectations, and the presence of soft-tissue damage^40, 55^. An attempt of repairing bone and cartilage defects by 3D scanning and 3D bioprinting was reported in this study. A precise 3D digital model was obtained rapidly and printed *in situ* perfectly using two kinds of photo-crosslinking hydrogels. With the development of correlative technology, this method provided a novel approach to treat open injuries in the skeletal system and might be more effective in some special cases.

## Materials and Methods

All methods in this study were carried out in accordance with relevant guidelines and regulations. All experimental protocols in this study were approved by the committee of Drum Tower Hospital affiliated to Medical School of Nanjing University.

### Data Availability

The datasets generated during and/or analysed during the current study are available from the corresponding author on reasonable request.

### Materials

Sodium alginate, Poly (ethylene glycol) Diacrylate (PEGDA), 2-Hydroxy-4′-(2-hydroxyethoxy)-2-methylpropiophenone (IrgacureTM 2959, as known as I-2959), N,N-methylenebis(acrylamide) (MBA), CaCl_2_, NaOH and methacrylic anhydride (MA) were purchased from Sigma Aldrich, USA. Sodium hyaluronic acid (HA) was purchased from Freda Biochem Co., Ltd. (Shandong, China). Water was ultrapure grade supplied from a Milli-Q purification system (Millipore, USA).

### Preparation of hydrogel

Alginate and HA hydrogel were prepared for printing using techniques described in previous studies^13, 16, 35^. The two solutions consisting of 6% w/v sodium alginate powder and 80 mM CaCl_2_ were mixed with a volume ratio of 1:1 to result in a partially cross-linked hydrogel. Then, 10% w/v PEGDA and 0.05% w/v I-2959 were added to provide a cytocompatible photoinitiating condition.

HA was modified with a double bond by reacting with the MA. 2 g of HA was dissolved in 100 mL of distilled (DI) water with stirring at 4 °C overnight, followed by addition of 1.6 mL of MA into the HA solution. The pH of the reaction was maintained between 8 and 9 by adding 5 N NaOH at 4 °C under continuous stirring for 24 h. Subsequently, m-HA was precipitated in acetone, washed with ethanol, and then dissolved in DI water. After dialysis against DI water for 48 h, the purified m-HA was obtained by lyophilization. Purified m-HA was dissolved in DI water to a final concentration of 5% w/v. I-2959 and MBA were added respectively at a concentration of 0.05% w/v and 1% w/v, respectively, to provide a cytocompatible photoinitiating condition. The viscosity of hydrogel was tested using a rotational viscometer (Brookfield, USA).

### Harvesting and preparation of the bone and cartilage

The bone and cartilage samples used for this study were harvested from a sacrificed 4-month-old New Zealand rabbit and a sacrificed 6-month-old Bama mini pig, respectively. Muscular and connective tissues were removed while maintaining the geometric integrity of the samples.

#### Creating of bone and cartilage defects

This study demonstrated three defects. The bone defect was created on a humeral bone of a New Zealand rabbit. An 8-mm-length cortical bone was removed to mimic large segmental defects of long bones. The cylindrical-shaped osteochondral defect with diameter of 6mm and depth of 3mm was created on a femoral condyle of a New Zealand rabbit. This defect was made to mimic a freeform fracture of femoral condyle in which both bone and cartilage tissues were injured. The chondral defect was created on a tibial plateau of a Bama mini pig. A full-thickness cartilage with an area of about 0.42 cm^2^ was removed to mimic International Cartilage Repair Society (ICRS) grade IV chondral lesion.

### 3D scanning and remodeling

A total of six samples, including three samples with defects and three healthy samples of the contralateral sides, were scanned by a 3D handheld scanner (EinScan-Pro, Shining 3D, China). A high-definition mode was chosen to complete the scanning process to obtain more accurate images. Photosensitive resin models of scanning data were fabricated by a digital light procession printer (DLP) (Prismlab, China) to compare with original samples for the purpose of verifying similarity.

The shapes of defects were remolded by Boolean operation through Magics 21 (Materialise, Belgium). Because the body is equipleural, the restoration data of the defects area were acquired by mirroring the corresponding healthy parts. The shapes of the defects could be achieved by matching and subtracting the STL files of injury and restoring in the Magics software.

### 3D bioprinting

A modified 3D printer (Bio-Architect, Regenovo, China) was used for this study. This instrument comprised four long-wave (365 nm) ultraviolet (UV) lamps for simultaneous photopolymerization during the printing. The UV intensity varied between 4 to 120 mw/cm^2^ according to the manufacturer, and the intensity of 100 mw/cm^2^ was verified using a UV light meter (KUHNAST-UV-365A, Germany). The hydrogel was printed and photopolymerized for each layer to repair defects in a layer-by-layer assembly. The viscosity of the hydrogel and the correlation printing parameter are shown in Table 2.

**Table 2:** Printing parameters.

Hydrogel	Viscosity (mPa s)	Needle (μm)	Air pressure (MPa)	Speed (mm/s)	Thickness (mm)
Alginate	2600	210	0.25	6.5	180
m-HA	3300	210	0.3	6	180

The samples were set in plastic foam, and the bottom of the defects was parallel to the printing platform. Hydrogel filaments were extruded by a nozzle with a diameter of 210 μm. The thickness of the scaffolds was set to 180 μm with θ = 0° and 90° in the slicing software (Regenovo, China). As the viscosity of alginate hydrogel was differ from that of m-HA hydrogel, the air pressure were set at 0.25 MPa and 0.3 MPa, respectively. The alginate hydrogel was used to repair the bone defect and osteochondral defect. The cartilage defect was repaired by m-HA hydrogel.

### Measuring printing accuracy

The volume of digital defect models were detected by Rhinoceros 5 (McNeel, USA). The samples were scanned again after printing, and the digital models were encapsulated by Geomagic Studio 12 (Geomagic, USA). The STL files of 3D printed samples and corresponding healthy parts were imported into Geomagic Qualify 12 (Geomagic, USA) and 3-matic STL 9.0 (Materialise, Belgium) to measure the accuracy by an operation named “3D Samples Comparison”. The full-color difference image was visualized and easy to determine the tolerance and fidelity of printed surface. Colors corresponded to error magnitude. The color of green represented that the two models can be fitted completely. The 3D error gradually increased as the color transferred from green to red, and gradually decreased as the color transferred to deep blue. The mean 3D error was calculated by Geomagic Qualify 12.
